# Acoustics-Augmented Diagnosis Method for Rolling Bearings Based on Acoustic–Vibration Fusion and Knowledge Transfer

**DOI:** 10.3390/s25165190

**Published:** 2025-08-21

**Authors:** Fangyong Xue, Chang Liu, Feifei He, Zeping Bai

**Affiliations:** 1 Key Laboratory of Advanced Equipment Intelligent Manufacturing Technology of Yunnan Province, Kunming University of Science &Technology, Kunming 650500, China; xuefy0812@163.com (F.X.); heff0523@163.com (F.H.); m13928994866@163.com (Z.B.); 2 Faculty of Mechanical & Electrical Engineering, Kunming University of Science &Technology, Kunming 650500, China

**Keywords:** acoustic–vibration fusion, heterogeneous transfer learning, knowledge distillation, acoustic diagnosis

## Abstract

Although contact-based vibration signal methods for mechanical equipment fault diagnosis demonstrate superior performance, their practical deployment faces significant limitations. In contrast, acoustic signals offer notable deployment flexibility due to their non-contact nature. However, acoustic diagnostic methods are susceptible to environmental noise interference, and fault samples are typically scarce, leading to insufficient model generalization capability and robustness. To address this, this paper proposes an acoustic–vibration feature fusion strategy based on heterogeneous transfer learning, further integrated with a knowledge distillation framework. By doing so, it aims to achieve efficient transfer of vibration diagnostic knowledge to acoustic models. In the proposed approach, a teacher model learns diagnostic knowledge from highly reliable vibration signals and uses this to guide the training of a student model on acoustic signals. This process significantly enhances the diagnostic capability of the acoustic-based student model. Experimental studies conducted on a custom-built test rig and public datasets demonstrate that the proposed method exhibits excellent diagnostic accuracy and robustness under unseen working conditions.

## 1. Introduction

The advent of the Industry 4.0 era has significantly propelled research and development in fault diagnosis methods for industrial equipment [1]. As critical components of rotating machinery, rolling-element bearings account for over 50% of total equipment failures [2] making their condition monitoring and diagnosis paramount. Online monitoring and fault diagnosis systems utilizing multiple physical quantities—such as acoustic, optical, electrical, vibrational, force, and temperature signals—are now extensively implemented in industrial environments. These systems enable the real-time acquisition of equipment condition information, substantially enhancing the production efficiency while reducing the operation and maintenance costs, thereby delivering considerable economic benefits to the manufacturing sector [3]. Among these, vibration-based condition monitoring detects approximately 90% of machine faults [4], serving as the primary method for bearing fault diagnosis. Vibration-based analysis methods capture structural dynamic responses via accelerometers, which offer high technical maturity in time-frequency feature extraction, along with good reliability and diagnostic accuracy. However, contact-based measurements face sensor installation constraints, significantly increasing the deployment difficulty in industrial scenarios such as high-temperature environments, confined spaces, or surfaces with oil contamination or corrosion.

To overcome the physical constraints in sensor deployment, non-contact intelligent monitoring systems have emerged as increasingly viable alternatives. For instance, Raouf et al. proposed a current signature analysis that circumvents direct sensor installation, enabling mechanical fault detection in robotic RV reducers [5] and bearing fault diagnosis in servo motors [6,7]. Sharma et al. employed non-invasive infrared thermography to characterize degradation states of bearing components, specifically inner/outer rings and rollers [8]. All these studies demonstrate efforts to transcend physical contact limitations. By contrast, acoustic signals offer unique advantages for fault information acquisition due to their non-contact measurement capability and vibro-acoustic coupling effects. The inherent coupling between acoustic and vibration signals generates both shared fault characteristics and complementary fault information, enabling the acquisition of richer diagnostic knowledge. Nevertheless, background noise in industrial sites and sound wave propagation attenuation effects result in a low signal-to-noise ratio (SNR) for fault features, constraining the diagnostic accuracy and generalization capability. This contradiction drives the evolution toward deep transfer diagnosis and multi-modal information fusion, where data fusion and knowledge transfer between vibration and acoustic signals provide a viable solution for enhancing diagnostic accuracy and reliability.

In bearing fault diagnosis research, traditional vibration-based methods grounded in time–frequency analysis exhibit high technical maturity, along with strong interpretability and versatility [9,10,11]. Concurrently, transfer learning-enabled intelligent diagnostics effectively mitigate challenges, including data scarcity [12], distribution discrepancy [13], and unidentified faults [14]. In contrast, acoustic signal-based diagnosis confronts more severe limitations. Conventional acoustic techniques (e.g., wavelet transforms [15], FFT, and correlation analysis [16]), despite their situational utility, demonstrate high sensitivity to environmental noise and susceptibility to multipath interference effects, encompassing signal reflection, scattering, and extraneous sound sources. These issues degrade the SNR of discriminative fault features, thereby constraining the diagnostic capability.

To address the challenges of the environmental noise interference and insufficient generalization in acoustic signals, current research primarily focuses on two directions: acoustic imaging/array technology and robust feature extraction. Acoustic imaging techniques based on microphone arrays, such as near-field acoustic holography (NAH) [17], the wave superposition method [18], and the 2.5-dimensional acoustic field spatial feature method [19], enhance signals and suppress noise through acoustic field reconstruction or spatial feature extraction and then combine support vector machines (SVM), random forests, or convolutional neural networks (CNNs) to improve the diagnostic accuracy. However, these methods often suffer from high feature dimensionality and large model parameter counts, making them difficult to deploy efficiently in real-world industrial settings that demand lightweight models. The robust feature extraction direction focuses on designing more noise-resistant algorithms to mine discriminative information from single-channel or simplified array signals. Representative works include using a concentric circular microphone array combined with direction-of-arrival (DOA) estimation to achieve noise suppression and target sound source enhancement while reducing hardware complexity [20]; and deep learning-based feature extraction frameworks, such as complex-domain dominant-frequency CNNs employing designed dominant-frequency filters to effectively suppress non-characteristic band noise [21] or combining supervised stacked denoising autoencoders with lightweight mobile vision transformers to learn noise-robust features from mel-frequency cepstral coefficients (MFCCs) [22]. These methods have demonstrated improved feature discriminability and model generalization in strong noise backgrounds. Nevertheless, the aforementioned studies primarily rely on single acoustic information, possessing inherent diagnostic limitations; furthermore, they mostly focus on stable operating conditions, leaving the challenges of diagnostic generalization and transfer learning under complex varying conditions largely unexplored.

Addressing the inherent limitations of single acoustic signal diagnosis and the generalization challenge under varying conditions, leveraging the complementarity and potential commonality in fault representation between vibration and acoustic signals becomes a key breakthrough direction for achieving acoustic–vibration signal fusion and knowledge transfer. Current research explores two parallel paths: complementary feature fusion (CFF) and common feature transfer (CFT). CFF exploits the synergistic advantages of vibration signals’ high sensitivity in the low-frequency range and acoustic signals’ abundant high-frequency details to construct robust fusion diagnostic models. Representative advances include the dynamic weighted multi-modal fusion mechanism proposed by Sun et al. [23], which adaptively adjusts weights to handle noise and small-sample challenges; the dual-scale time–frequency attention network developed by Yan et al. [24], effectively fusing time–frequency features of heterogeneous data via signal-image transformation and time–frequency attention mechanisms; and the residual attention-guided vision transformer utilized by Lian et al. [25] to integrate complementary acoustic–vibration features, significantly enhancing performance under both constant and variable speeds. Such fusion methods typically depend on the effective installation of vibration sensors, facing fundamental constraints in industrial scenarios where contact-based measurement is difficult. CFT research aims to uncover the underlying common fault representations between vibration and acoustic signals, enabling knowledge transfer to alleviate the data scarcity and distribution shifts in the target signal, particularly acoustics. For instance, Chen et al. [26] employed multi-modal self-supervised pre-training to capture transferable robust features for single-sample diagnosis of cross-condition acoustic data; Gianpio et al. [27] explored transfer strategies using acoustically pre-trained models fine-tuned on vibration samples; Zhang et al. [28] proposed a fine-tuning-based convolutional recurrent neural network method to transfer diagnostic knowledge from vibration data to acoustic diagnosis models; and Wu et al. [29] introduced the MD3VA method, combining fault feature enhancement and generative adversarial networks (GANs) to learn fault distributions from the vibration domain and generate high-fidelity acoustic virtual samples, addressing target domain sample scarcity for cross-equipment transfer. Challenges in CFT methods remain: the underlying feature distributions of vibration and acoustic signals differ significantly, making transfer strategies based on simple fine-tuning often inadequate to bridge the semantic gap between modalities effectively; GAN-based techniques for generating acoustic fault virtual samples commonly face instability during training and the risk of mode collapse, impacting the sample diversity and fidelity.

To address the inherent limitations of single acoustic diagnosis and the challenges in existing acoustic–vibration common knowledge transfer methods, this paper proposes an acoustics-augmented diagnosis method for rolling bearings based on acoustic–vibration fusion and knowledge transfer. The main research encompasses the following aspects: A super-large-kernel lightweight convolutional neural network (SLKNet) is proposed. Its super-large-kernel design is tailored to meet the requirement for deep feature mining from both vibration and acoustic signals, while offering the advantages of lightweight architecture and ease of engineering deployment. Subsequently, an acoustic–vibration feature fusion method based on symmetric feature transfer theory is introduced. This method formulates the fusion problem of heterogeneous acoustic and vibration features as an optimization task for a common deep feature subspace Z. A joint knowledge distillation network is designed that integrates intermediate-layer feature alignment with output-layer knowledge distillation. This fusion enables vibration information to guide the condensation of acoustic knowledge. Operating within a knowledge distillation transfer framework, this strategy facilitates the transfer of diagnostic knowledge from a vibration-trained teacher model, thereby enhancing the acoustically trained student model’s learning of fault knowledge and improving the acoustic diagnostic performance through augmentation with high-quality vibration knowledge. Finally, an acoustic diagnosis experimental scheme is designed to validate the diagnostic performance of the proposed acoustics-augmented model under variable load conditions. The main contributions of this paper are summarized as follows:

(1) SLKNet is proposed, employing a super-large-kernel size of 160. Serving as the core model for acoustic–vibration signal feature extraction, its super-large-kernel significantly enhances global fault feature capture capability, while the lightweight design ensures efficient deployment in industrial scenarios.
(2) Given that research on the heterogeneous transfer between vibration and acoustic signals remains relatively insufficient, an acoustic–vibration feature fusion method based on symmetric feature transfer theory is introduced. Utilizing SLKNet as the backbone network to construct dual-path feature mapping, this method drives feature distribution alignment and enforces semantic consistency constraints within a shared subspace, effectively bridging the discrepancies arising from physical dimensions and feature distributions. This feature-level transfer strategy offers a novel solution perspective for vibration–acoustic cross-modal transfer, fundamentally distinct from existing paradigms of model tuning or data generation.
(3) Building upon the proposed acoustic–vibration fusion method, a vibration–acoustic knowledge distillation transfer pathway is constructed by exploring the intrinsic consistency between the knowledge distillation framework and symmetric feature mapping theory, establishing it as the core pathway for cross-modal knowledge transfer. Through joint distillation, deep abstract features from the intermediate layers and discriminative knowledge from the output layer of the vibration-trained teacher model are transferred to the acoustically trained student model. This process achieves deep guidance of vibration information over acoustic fault knowledge—an innovative application of distillation technology in the field of vibration–acoustic knowledge enhancement.
(4) Comprehensive validation experiments and evaluation methods for acoustic–vibration knowledge transfer are designed. This includes a transfer benchmark based on the maximum mean discrepancy (MMD) alignment, covering both acoustic-to-acoustic transfer and vibration-to-acoustic transfer scenarios to provide a rigorous reference for cross-modal gain evaluation. Furthermore, cross-condition deep feature space visualization is introduced to intuitively reveal the model transferability and robustness.

The main contents of this paper are structured as follows: Section 2 describes the fundamental theories. Section 3 details the construction process of the proposed model and methods. Section 4 validates the superiority of the proposed approach through two experimental case studies. Section 5 discusses the conclusions and perspectives.

## 2. Related Works

### 2.1. Feasibility Study of Acoustic–Vibration Transfer

The homologous nature of acoustic and vibration signals forms the foundation for their information fusion and guarantees the feasibility of knowledge transfer and acoustic diagnosis enhancement. The periodic motion of rotating machinery propagates through solid media as mechanical vibrations, while manifesting as airborne acoustic signals at the same frequency through air [30]. Fundamentally, both signals originate from identical excitation sources but represent distinct energy manifestations due to their propagation through different physical media. According to the “source–path–receiver” model [31], vibration and noise represent homologous but heterogeneous state information. Their differences stem from the physical characteristics of the propagation media and receiving sensors: vibration sensors capture structural responses via solid contact, while microphones acquire airborne sound waves non-contactively. Despite differing acquisition modes, both signals can be converted into time-varying electrical signals, establishing a unified basis for time–frequency analysis.

The core commonality lies in the fact that both vibration and acoustic signals carry characteristic information about the equipment’s operating state. This information homology provides the physical basis for cross-modal transfer. Conversely, differences in signal attenuation characteristics and SNR distributions, caused by distinct propagation paths, result in modality-specific features. This homologous yet heterogeneous characteristic implies that high-SNR diagnostic knowledge from the vibration domain can be transferred to the acoustic domain. This provides the theoretical foundation for circumventing the limitations of contact-based measurement.

Wu et al. [29] mathematically characterized the homologous yet heterogeneous nature of vibration and acoustic signals. The signal acquired by a vibration sensor follows this mathematical formulation:

(1) $$x_{V}(t)=[x_{V1}(t)+x_{V2}(t)]*h_{V}(t),$$

where $x_{V1}(t)$ represents the fault signal component, $x_{V2}(t)$ represents the component from normal equipment operation, and $h_{V}(t)$ denotes the transmission path of the vibration signal within the mechanical structure.

Correspondingly, the signal acquired by an acoustic sensor is expressed as

(2) $$x_{A}(t)=\left\{\left[x_{A1}(t)+x_{A2}(t)]*h_{AS}(t)+N_{A}(t\right)\right\}*h_{A}(t),$$

where $x_{A1}(t)$ and $x_{A2}(t)$ represent the fault signal component and the normal operation signal component, respectively, $h_{AS}(t)$ and $h_{A}(t)$ denote the transmission path within the structure and the airborne propagation path for the acoustic signal, respectively, and $N_{A}(t)$ represents the environmental noise.

Based on the theoretical analysis of the acoustic–vibration signal relationship and this mathematical modeling, the present study establishes the following hypotheses for conducting heterogeneous cross-modal transfer diagnosis using knowledge distillation:

(1) Homology of fault knowledge hypothesis:

(3) $$x_{V1}(t)\approx x_{A1}(t)$$

This hypothesis constitutes the fundamental basis for the proposed acoustic–vibration fusion framework.

(2) Feature projection hypothesis:

(4) $$f(x_{V1}(t))\approx f(x_{A1}(t))$$

This hypothesis underpins the knowledge transfer mechanism within the proposed knowledge distillation framework.

### 2.2. Transfer Learning

Transfer learning has emerged in recent years as a promising approach to address machinery fault diagnosis under varying working conditions. It enhances task performance in the target domain by reusing knowledge learned from the source domain. Its core challenge lies in resolving discrepancies in the feature space or data distribution between the source and target domains. Within mechanical fault diagnosis, this technology effectively mitigates challenges such as high data annotation costs and difficulties in modeling varying working conditions [32,33].

The theoretical foundation of transfer learning rests on rigorously defined concepts of domain and task [34]. A domain D={X,P(x)} consists of a feature space X and a marginal probability distribution P(x), where $x=\{x_{1},\cdots ,x_{n}\}\in X$. A task T={Y,f} comprises a label space Y and a predictive function f, which is equivalent to P(y|x).

When the feature spaces are isomorphic $\left(X_{s}=X_{t}\right)$ and the label spaces are consistent $\left(Y_{s}=Y_{t}\right)$, but distribution discrepancy exists, it constitutes homogeneous transfer learning. Here, the focus is solely on addressing data distribution differences. Conversely, when the feature spaces of the source and target domains are intrinsically different $\left(X_{s}=X_{t}\right)$, regardless of label space consistency $\left(Y_{s}\neq Y_{t}\right)$ or inconsistency $\left(Y_{s}=Y_{t}\right)$, it falls under the category of heterogeneous transfer learning [35]. Heterogeneous transfer learning is particularly crucial in mechanical fault diagnosis, such as when handling heterogeneous sensing data like acoustic and vibration signals.

The core challenge in heterogeneous transfer learning is bridging the semantic gap between the source and target domains, stemming from the intrinsic heterogeneity of their feature spaces. Existing methods primarily employ symmetric and asymmetric feature transfer strategies. Among these, symmetric feature transfer methods are widely adopted due to their higher prediction accuracy [36]. The core principle involves constructing dual mapping functions to align the heterogeneous feature spaces into a shared subspace, thereby ensuring that instances from the source and target domains are closer within this subspace to support effective knowledge transfer.

Within the theoretical framework of symmetric feature transfer, the source domain features are first mapped:

(5) $$D_{s}^{\prime }=f_{V}(D_{s}),$$

where $x_{s}^{\prime }=f_{V}(x_{s})$. Similarly, the target domain features are mapped:

(6) $$D_{t}^{\prime }=f_{A}(D_{t}),$$

where $x_{t}^{\prime }=f_{A}(x_{t})$. Here, $f_{V}$ and $f_{A}$ represent the learnable mapping operators for the vibration source domain and acoustic target domain, respectively. Within the shared subspace Z, the optimization process achieves dual distribution alignment by minimizing the following objective function:

(7) $$\min_{f_{V},f_{A}}D_{f}\left[P\left(x_{s}^{\prime }\right),P\left(x_{t}^{\prime }\right)\right]+\lambda D_{f}\left[P\left({\hat{y}}_{s}^{\prime }|x_{s}^{\prime }\right),P\left({\hat{y}}_{t}^{\prime }|x_{t}^{\prime }\right)\right],$$

where $D_{f}[\cdot ]$ denotes a distribution metric function; $P\left(x^{\prime }\right)$ represents the marginal probability distribution after mapping; $P\left({\hat{y}}^{\prime }|x^{\prime }\right)$ represents the conditional probability distribution after mapping; and λ is an explicit loss balancing factor.

Through this optimization, the subspaces $D_{s}^{\prime }$ and $D_{t}^{\prime }$ are constrained to belong to Z, and the inter-domain distribution discrepancy is minimized. This enhances the effectiveness of knowledge transfer.

### 2.3. Knowledge Distillation

Knowledge distillation (KD) is a model compression and knowledge transfer technique. Its core objective is to effectively transfer the “knowledge” learned by a high-performance but complex teacher model to a structurally simpler and computationally lighter student model. Initially proposed by Hinton et al. [37] to address model compression challenges, KD demonstrated significant efficacy in tasks like image classification and has since been extended to various domains, including fault diagnosis [38,39]. The core value of KD lies in its teacher–student paradigm, enabling the student model to learn not only the label information from the original training data but also to mimic the “soft targets” generated by the teacher model. These soft targets encapsulate richer distributional information, significantly enhancing the student model’s generalization capability and final performance. This mechanism is particularly advantageous for lightweight model deployment or scenarios where a powerful model guides a weaker one.

Unlike standard neural networks that typically use a “softmax” output layer to produce class probabilities, KD introduces the concept of soft targets. This layer is modulated by a temperature parameter *T*, converting the logits $z_{i}$ for each class into normalized probability values $q_{i}$:

(8) $$q_{i}=\frac{exp(z_{i}/T)}{\sum_{j}exp(z_{j}/T)}.$$

The standard setting for the temperature parameter *T* is 1. Increasing *T* yields a smoother class probability distribution.

The teacher model is trained using the standard cross-entropy loss with *T* = 1:

(9) $$L_{teacher}=-\sum_{i}y_{i}log(p_{i}),$$

where $y_{i}$ is the one-hot encoded true label, and $p_{i}$ is the output probability of the teacher model at temperature *T* = 1.

The student model’s loss combines two components:

(1) Hard loss: the cross-entropy loss with the true labels at *T* = 1.
(2) Soft loss: the Kullback–Leibler (KL) divergence loss between the softened output distributions of the teacher and student models, computed at temperature *T*.

(10) $$L_{student}=(1-\alpha )\underset{Hard\;Loss}{\underset{︸}{\left(\sum_{i}-y_{i}log(q_{i})\right)}}+\alpha T^{2}\underset{Soft\;Loss}{\underset{︸}{\left(\sum_{i}p_{i}^{T}log\frac{p_{i}^{T}}{q_{i}^{T}}\right)}},$$

where α is the weighting coefficient balancing hard and soft losses, $T^{2}$ scales the KL divergence term, and $p_{i}^{T}$ and $q_{i}^{T}$ represent the soft target probability distributions from the teacher and student models respectively at temperature *T*.

## 3. Proposed Methodology

### 3.1. Transfer Diagnosis Scenario

**Figure 1:**
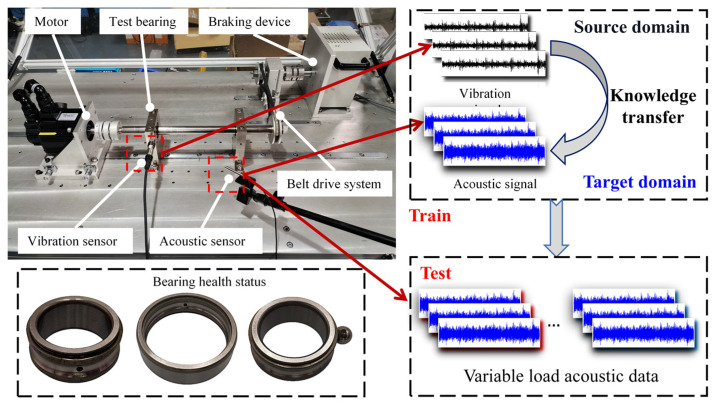
Transfer diagnosis scenario.

Leveraging the non-contact acquisition capability of acoustic signals overcomes the installation limitations of vibration sensors. However, strong background noise and propagation attenuation constrain the diagnostic accuracy and generalization capability, posing a key challenge for practical industrial monitoring. To address this, this study designs a transfer diagnosis scenario under varying working conditions, as depicted in Figure 1.

This scenario utilizes a contact accelerometer and a non-contact acoustic sensor to collect vibration signals and acoustic signals, respectively. To effectively extract fault information in the frequency domain, the acoustic signals undergo pre-emphasis processing. Subsequently, both signal types are converted into amplitude spectra. Within this cross-domain framework, the vibration signals constitute the source domain, while the acoustic signals define the target domain. First, a teacher model is trained using the vibration data. This teacher model then guides the learning process of the acoustic student model. This transfer scenario aims to achieve knowledge transfer from the vibration domain to the acoustic domain, ultimately enhancing the diagnostic robustness of the acoustic model in noisy environments and under variable load conditions.

### 3.2. Super-Large-Kernel Lightweight Convolutional Neural Network

**Figure 2:**
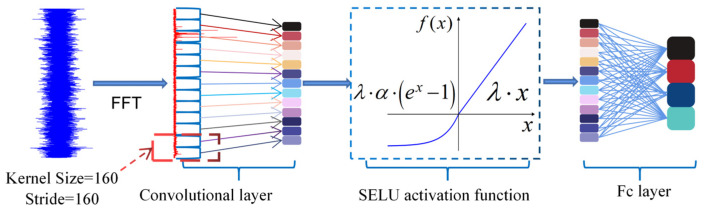
The structure of the proposed SLKNet.

To extract fault-discriminative knowledge from acoustic–vibration signals in deep feature spaces, this study proposes the super-large-kernel lightweight convolutional neural network (SLKNet), as illustrated in Figure 2.

The SLKNet adopts a two-stage architecture comprising a feature extraction stage and a classification stage to capture global fault information embedded in input spectra while addressing deployment constraints on edge devices. The feature extraction stage utilizes a single-layer super-large convolutional kernel with a size of 160 and stride of 160, enabling broad receptive fields to enhance global signal perception. Nonlinear feature representation is enhanced via scaled exponential linear units (SELU), while the classification stage employs fully connected layers. Compared to traditional deep CNN, SLKNet reduces the model depth and parameters through its single-layer super-large-kernel design. This lightweight structure retains robust global feature extraction capabilities, meeting efficiency requirements for industrial edge deployment.

### 3.3. Acoustic–Vibration Feature Fusion Method

**Figure 3:**
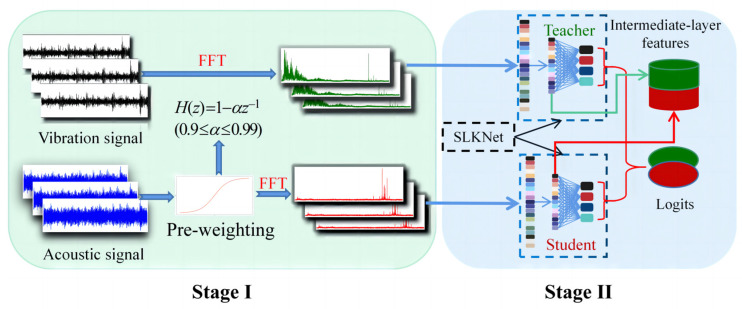
Proposed two-stage acoustic–vibration feature fusion method.

To address the heterogeneous transfer challenge between vibration and acoustic signals, this study proposes a two-stage feature fusion method. Its workflow is illustrated in Figure 3.

Stage I undertakes explicit feature alignment and primary fusion. Acoustic signals exhibit high-frequency attenuation that weakens the key impact features of bearing faults (e.g., pitting, cracks). To enhance these components, a pre-emphasis process [22] is applied for high-frequency energy compensation. Pre-emphasized acoustic signals and raw vibration signals are then uniformly transformed into amplitude spectra via the fast Fourier transform. This frequency-domain mapping simultaneously corrects physical deviations in acoustic signals through pre-emphasis compensation and unifies feature representations within the spectral domain, thereby mitigating the feature space mismatch and physical unit inconsistency induced by the measurement divergence.

Stage II progresses to implicit feature alignment and advanced fusion via deep modeling. While both vibration and acoustic signals contain fault-related information, their inherent differences in physical origin and feature space distribution create a fundamental heterogeneity. This prevents the direct transfer of features learned from the vibration source domain to the acoustic target domain, constituting a heterogeneous feature transfer problem. To address this challenge of acoustic–vibration heterogeneous feature transfer, Stage II employs symmetric feature transfer theory. It establishes a cross-domain feature mapping mechanism, transforming the heterogeneous feature alignment problem into an optimization task for a shared deep feature subspace, Z. Specifically, leveraging the powerful feature extraction capability of the SLKNet network, the spectral features unified in Stage I are further projected into this common subspace Z. SLKNet extracts both intermediate-layer features and decision-level features from vibration and acoustic signals. Guided by symmetric transfer theory, these features are projected into the shared subspace Z, enabling deep mining and fusion of cross-modal fault knowledge. This process achieves deep-level feature alignment and fusion. More crucially, it establishes a theoretical foundation for the effective transfer of knowledge from the vibration source domain to the acoustic target domain and the enhancement of diagnostic performance.

### 3.4. Constructing the Vibration–Acoustic Knowledge Distillation Transfer Pathway

To achieve effective knowledge transfer from vibration to acoustics, this paper introduces a knowledge distillation (KD) framework to construct the diagnostic knowledge transfer pathway. The suitability of this method manifests in two key dimensions. Firstly, vibration signals, which contain high-quality fault features but face installation constraints, naturally align with the teacher role. Conversely, the acoustic diagnosis model, characterized by feature ambiguity, requires guidance through the student role. Secondly, the inherent suitability of the knowledge distillation teacher–student paradigm stems from its essential isomorphism with symmetric feature mapping theory, as both frameworks project heterogeneous feature spaces onto a common subspace Z. Specifically, the KD process can be formulated as a dual-space mapping problem, which is expressed as follows.

The teacher feature space mapping corresponds to source domain feature mapping:

(11) $$D_{s}^{\prime }\leftarrow D_{Teacher}^{\prime }=f_{V}^{Teacher}(D_{Teacher}).$$

The student feature space mapping corresponds to target domain feature mapping:

(12) $$D_{T}^{\prime }\leftarrow D_{Student}^{\prime }=f_{A}^{Student}(D_{Student}),$$

where $f_{A}$ and $f_{A}$ are the learnable parameter matrices of the teacher and student models, respectively.

Based on this theory, processing vibration signals through the teacher model maps its intermediate-layer abstract features and output-layer discriminative knowledge to the shared feature subspace $Z_{middle}^{T}$ and discriminative space $Z_{logit}^{T}$. Correspondingly, processing acoustic signals through the student model yields $Z_{middle}^{S}$ and $Z_{logit}^{S}$. Consequently, the acoustic–vibration feature fusion problem transforms into designing the teacher–student model architecture and optimizing the corresponding loss functions.

Identical but parameter-independent SLKNet architectures are employed to construct the teacher and student models. Their structural similarity reduces the difficulty of cross-modal transfer. Deep transfer is realized through a joint strategy combining intermediate-layer feature alignment and output-layer distillation.

#### 3.4.1. Intermediate-Layer Feature Alignment

Alignment is performed using the flattened features $H_{t}\in {\mathbb{R}}^{B\times D}$ and $H_{s}\in {\mathbb{R}}^{B\times D}$ output from the convolutional layers of the teacher and student models, respectively, where *B* is the batch size, and *D* is the feature dimension. Mean pooling along the feature dimension yields the compressed vector representations:

(13) $$\left\{\begin{array}{l} z_{t}^{\left(b\right)}=\frac{1}{D}\sum_{d=1}^{D}H_{t}^{\left(b,d\right)}\\ z_{s}^{\left(b\right)}=\frac{1}{D}\sum_{d=1}^{D}H_{s}^{\left(b,d\right)} \end{array}\right..$$

The negative cosine similarity is adopted as the intermediate-layer feature alignment loss function:

(14) $$L_{mid}=1-\frac{\langle z_{t},z_{s}\rangle }{\|z_{t}{\|}_{2}\cdot \|z_{s}{\|}_{2}},$$

where 〈⋅,⋅〉 denotes the inner product between vectors, and $\|\cdot {\|}_{2}$ denotes the $L_{2}$ norm.

#### 3.4.2. Output-Layer Knowledge Transfer

The temperature coefficient τ softens the raw output $y_{t}$ of the teacher model:

(15) $$q_{t}=softmax\left(\frac{y_{t}}{t}\right).$$

Knowledge transfer is achieved by minimizing the KL divergence between the student output probability $q_{s}$ and $q_{t}$:

(16) $$L_{out}=KL(q_{t}\|q_{s}).$$

The total loss combines both components:

(17) $$L_{total}=L_{out}+aL_{mid},\;\alpha \in (0,1),$$

where α is a hyperparameter regulating the contribution of the intermediate-layer loss.

This method overcomes the limitations of traditional parameter transfer. By transferring probability distributions and abstract feature representations, it effectively bridges the semantic gap between heterogeneous modalities. Vibration knowledge, transferred to the acoustic domain via this distillation pathway, significantly enhances the generalization capability and robustness of the acoustic diagnosis model, providing a viable technical pathway for aligning heterogeneous feature spaces.

### 3.5. Acoustic–Vibration Knowledge Transfer Diagnosis Method Based on Joint Knowledge Distillation

To overcome the deployment challenges of vibration sensors and address the limitations inherent in acoustic-based diagnostic models, including their susceptibility to noise interference, the scarcity of fault samples, and consequently constrained generalization capability, this study proposes an acoustic–vibration knowledge transfer diagnostic method based on joint knowledge distillation (JKD-AV). This method aims to transfer the high-quality fault feature knowledge embedded within vibration signals to acoustic signal diagnostic tasks, thereby enhancing the performance of acoustic data-based fault diagnosis models under variable load conditions.

**Figure 4:**
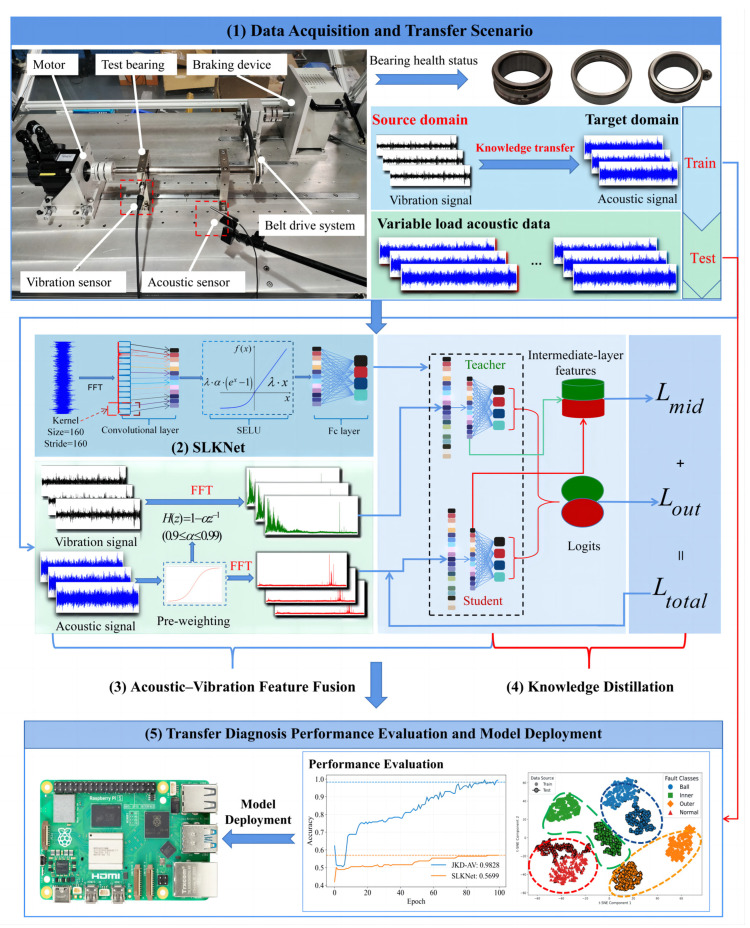
Overall framework of the JKD-AV method.

The overall framework of the proposed method is illustrated in Figure 4, with the specific steps detailed below:

(1) Collecting vibration and acoustic signals from bearings under multiple loads and health states to establish cross-condition transfer tasks.
(2) Implementing SLKNet as the unified backbone for feature extraction and knowledge fusion.
(3) Applying pre-emphasis to acoustic signals for high-frequency compensation, converting all signals to amplitude spectra via FFT, and mapping them to a shared subspace Z through SLKNet’s dual branches to fuse cross-modal features.
(4) Constructing vibration-based teacher models and acoustic-based student models using parameter-independent SLKNet instances and transferring vibration knowledge via joint optimization of feature-layer cosine alignment loss $L_{mid}$ and output-layer KL divergence loss $L_{out}$.
(5) Evaluating the student model on cross-load acoustic test sets using accuracy, confusion matrices, and t-SNE visualizations, with benchmarking against MMD-based transfer learning. Subsequently, the enhanced acoustic model, based on vibration transfer, was deployed on a Raspberry Pi platform. This lightweight deployment aimed to validate the model and benchmark its real-time inference performance for edge computing.

## 4. Experimental Validation

Experimental validation was conducted using a self-collected dataset and the BJTU-RAO bogie dataset. The detailed experimental configurations and procedures are described herein. The computing platform comprised an Intel Core i9-13900HX processor, NVIDIA RTX 4060 GPU, 32 GB DDR5 RAM, Windows 11 OS, and PyTorch 3.11 framework.

### 4.1. Case Study I: Self-Collected Experimental Bearing Dataset

#### 4.1.1. Dataset Description

**Figure 5:**
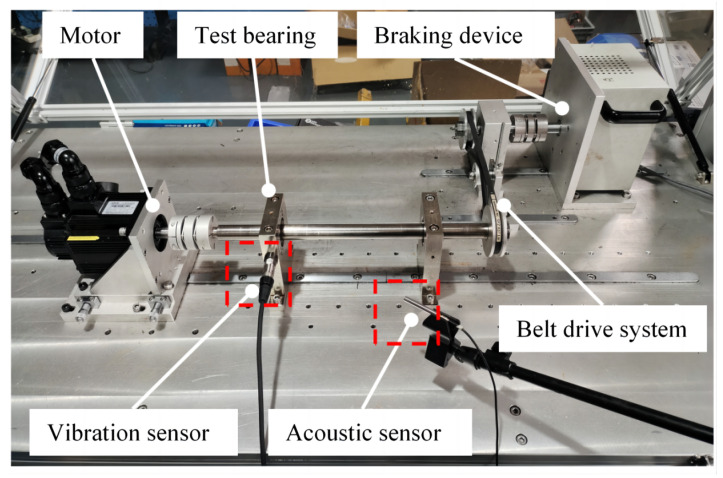
Fault simulation test rig.

The self-collected dataset was acquired from a fault simulation test rig illustrated in Figure 5. The rig included a three-phase induction motor, dual-support bearing housings, a belt drive system, and a magnetic powder brake. The test specimens comprised 6205 deep-groove ball bearings, with artificially damaged bearings installed in the left housing and healthy bearings in the right housing.

**Figure 6:**
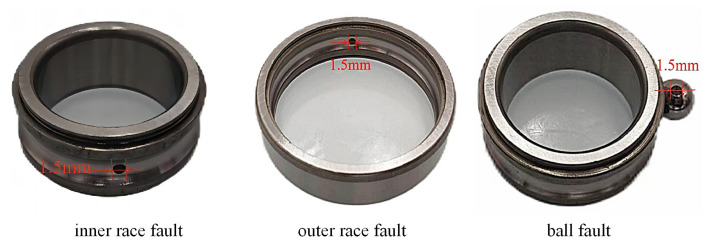
Three types of bearing failure conditions at a damage level of 1.5 mm.

Fault types were simulated via electrical discharge machining, introducing cylindrical pits (diameter: 1.5 mm, depth: 0.5 mm) on inner rings, outer rings, and rolling elements. This yielded four health states: normal, inner race fault, outer race fault, and ball fault, as depicted in Figure 6.

**Figure 7:**
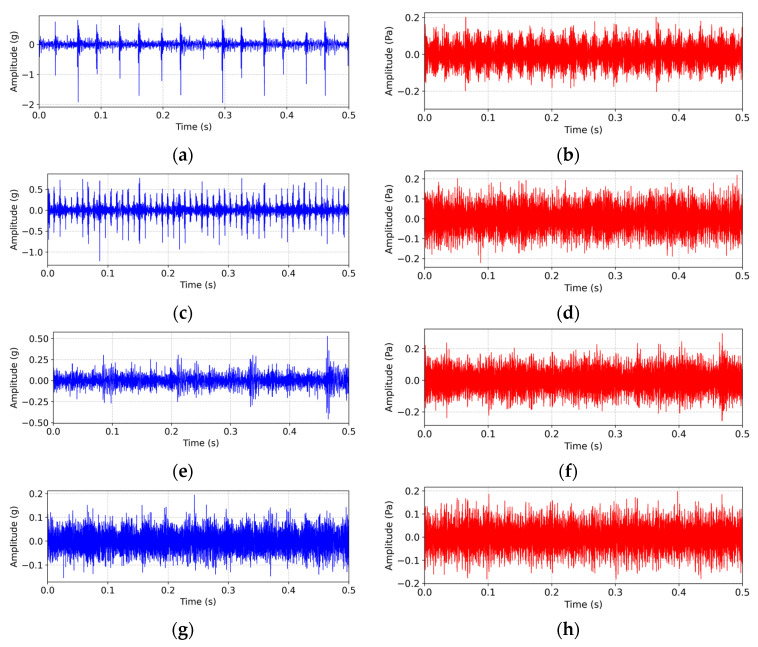
Vibration (blue) and acoustic (red) waveforms under 0 Nm working conditions. (**a**,**b**) Inner race fault; (**c**,**d**) outer race fault; (**e**,**f**) ball fault; (**g**,**h**) normal state.

An accelerometer was mounted radially on the left bearing housing. Synchronously, acoustic signals were captured by a microphone positioned 20 cm from the housing at a 45° angle. The sampling frequency was 25.6 kHz. Experiments were performed at a constant speed of 1800 rpm under three load conditions: 0 Nm, 3 Nm, and 6 Nm. Each condition yielded 15 s of continuous recordings, generating 384,000 data points per vibration/acoustic signal. Figure 7 displays raw waveforms acquired under 0 Nm loading. The vibration signals (blue traces) exhibit distinct fault characteristics: periodic impacts in inner race faults (a), modulated envelopes in outer race faults (c), quasi-periodic fluctuations in ball faults (e), and stable oscillations in healthy states (g). This confirms their high sensitivity to mechanical impacts. Conversely, the acoustic signals (red traces) demonstrate severe noise contamination. Only the inner race faults (b) retain discernible impact signatures, while other conditions—outer race (d), ball (f), and healthy (h) states—exhibit high waveform similarity. Critically, matching impact periodicity observed in both modalities verifies the inherent fault information presence, though acoustic signals suffer significant attenuation through propagation losses and ambient noise interference.

**Figure 8:**
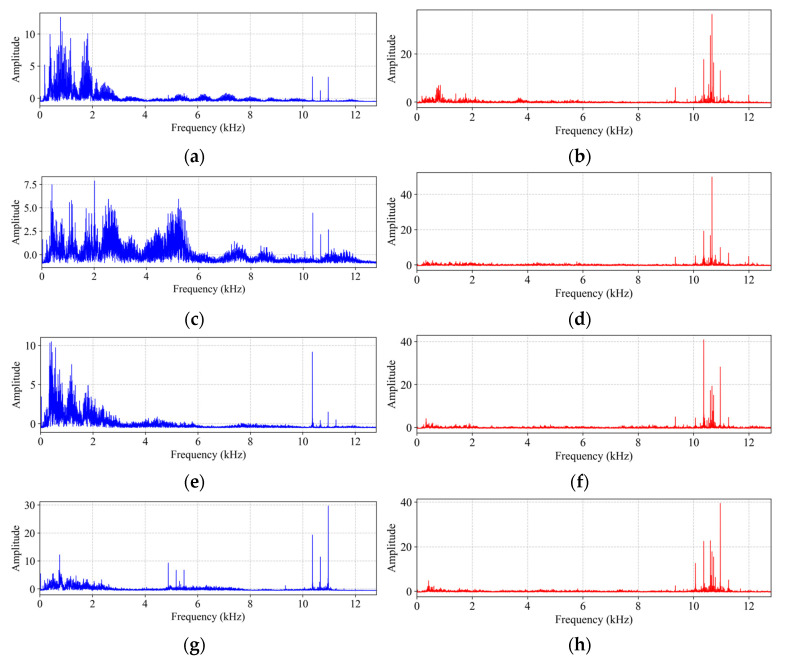
Vibration (blue) and acoustic (red) single-sided amplitude spectra under 0 Nm working conditions. (**a**,**b**) Inner race fault; (**c**,**d**) outer race fault; (**e**,**f**) ball fault; (**g**,**h**) normal state.

For model training, the raw waveforms were converted to single-sided amplitude spectra. The signals were evenly sampled, pre-emphasized for acoustic compensation, standardized, and transformed via FFT. Figure 8 presents the spectra under 0 Nm. The vibration spectra demonstrated clear fault discriminability across 0–12 kHz, with significant differences in the energy distribution, resonance peaks, and amplitude among states. The acoustic spectra suppressed the low-frequency noise post-pre-emphasis, concentrating the energy above 8 kHz. Critically, within the 10–12 kHz band, the vibration and acoustic spectra demonstrated pronounced synergistic coherence, characterized by the exact alignment of resonance peaks. This confirms effective deep fault feature extraction from preprocessed acoustics and reveals a shared high-frequency physical response mechanism.

The self-collected dataset comprised vibration and acoustic signals from four health states. The data were categorized by fault type and partitioned into three subsets by the load condition: Set A, Set B, and Set C. Each subset contained matched vibration–acoustic pairs with consistent labels, randomly shuffled. The dataset composition is summarized in Table 1.

A domain adaptation framework evaluates the cross-condition transfer capability. One load condition is designated as the training set, comprising both vibration and acoustic modalities to facilitate cross-modal knowledge transfer. Another condition is partitioned in a 1:9 ratio into validation and test sets. The validation set is utilized for hyperparameter tuning, while the test set assesses the generalization capability to unseen operating conditions. The configuration of the transfer tasks is detailed in Table 2.

#### 4.1.2. Experimental Results

To systematically evaluate the effectiveness of the proposed joint knowledge distillation-based acoustic–vibration method (JKD-AV), three transfer tasks were designed: A → B, A → C, and B → C. The acoustic baseline method SLKNet without knowledge distillation served as the comparative benchmark. The experimental results demonstrate the superior performance of the JKD-AV across all tasks, achieving diagnostic accuracies of 99.58%, 97.85%, and 97.49%, respectively. Compared to SLKNet, JKD-AV improved the accuracy by 5–30%, significantly enhancing the generalization capability of acoustic signals under complex operating conditions.

**Figure 9:**
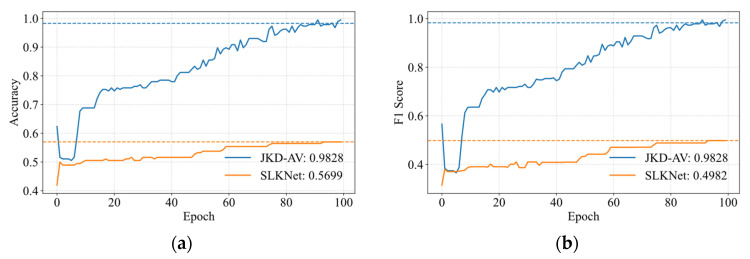
Validation set optimization convergence characteristics for task A → C transfer. (**a**) Validation accuracy; (**b**) F1-score.

Figure 9 illustrates the optimization mechanism of the JKD-AV through validation curves for task A → C. The validation accuracy and F1-score exhibit a consistent upward trend with increasing epochs, ultimately converging to peak values. The dashed lines in the figures represent the average results of the last five epochs on the validation set during training, and their specific values are shown in the legend. The JKD-AV substantially outperforms the SLKNet baseline in both metrics.

**Figure 10:**
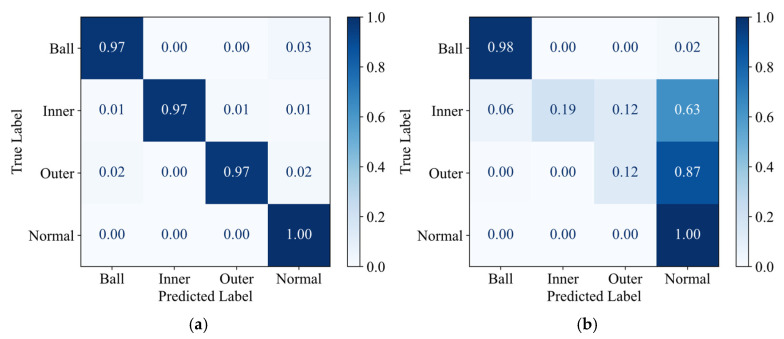
Confusion matrices for task A → C transfer. (**a**) JKD-AV; (**b**) SLKNet.

To validate the optimization of the classification boundaries by knowledge distillation, confusion matrices and t-SNE visualizations of deep features from the fully connected layer were analyzed for task A → C. Figure 10a clearly demonstrates the superior overall classification performance of the JKD-AV method. The recognition accuracy for all four fault classes exceeds 97%, indicating its successful learning of highly discriminative feature representations distinguishing the various fault types. In contrast, Figure 10b reveals that the SLKNet method can only effectively differentiate between rolling element faults and the normal state class. Significant deficiencies are observed in identifying both inner and outer race faults, exhibiting high misclassification rates (81% and 88%, respectively), primarily involving confusion with the normal state samples. The comparative results indicate that the knowledge distillation process effectively guides the student model to transfer more discriminative essential fault characteristics learned from the teacher model trained on high SNR vibration signals. This consequently leads to a significant enhancement in the robustness and generalization capability of the student model when processing acoustic signals.

**Figure 11:**
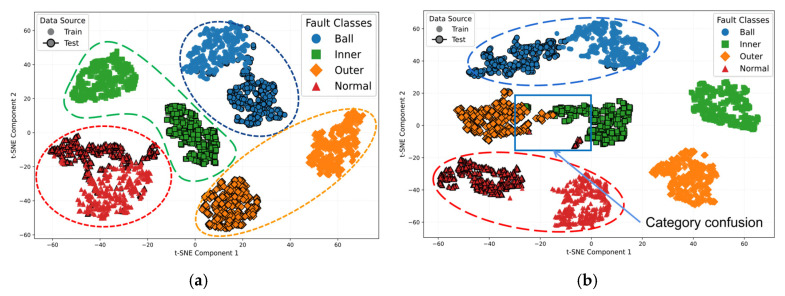
t-SNE visualizations for task A → C transfer. (**a**) JKD-AV; (**b**) SLKNet.

To analyze the impact of knowledge distillation on the feature space structure, deep features from the training and test sets were concatenated and labeled by source. t-SNE dimensionality reduction generated low-dimensional embeddings, as shown in Figure 11. The training samples are denoted by solid markers without borders; the test samples are marked with solid black borders. When feature distributions between training and test sets are similar, samples of the same class form compact clusters or localized overlaps after dimensionality reduction. Significant distribution divergence causes spatial dispersion, impeding cluster formation, disrupting spatial continuity, and preventing distinct decision boundaries.

Figure 11a demonstrates that in the JKD-AV method, the training and test set feature distributions of ball fault and normal state samples exhibit significant overlap and fusion, forming compact clusters with well-defined boundaries. Although the inner race and outer race faults display no substantial overlap in feature distributions between their training and test sets, each develops independent decision boundaries (indicated by orange and green dashed lines) without interference from other categories. This indicates that the features acquired through knowledge distillation maintain structural consistency in low-dimensional space, where minimal distributional discrepancy between the training and test sets is observed concurrently with high inter-class separability, demonstrating the method’s exceptional generalization capability.

In contrast, Figure 11b reveals that the SLKNet method exhibits highly overlapping and intertwined feature distributions for inner race and outer race faults, resulting in severely blurred class boundaries. Conspicuous dispersion of training and test set features is observed, with intra-class samples demonstrating sparse spatial distributions. The continuous decision boundary formation is impeded as evidenced by the interruption of the outer race fault’s orange cluster by the inner race fault test set’s green cluster.

Comparative analysis of Figure 11a,b demonstrates that JKD-AV exhibits superior inter-set alignment, higher intra-class compactness, and clearer inter-class separability. In contrast, SLKNet displays pronounced inter-class confusion and inter-set feature dispersion. These feature space disparities collectively validate the efficacy of knowledge distillation in optimizing the feature representations, enhancing the distribution consistency, and improving the cross-modal fault diagnosis generalization, significantly strengthening the acoustic-based rolling bearing fault diagnosis capability.

#### 4.1.3. Ablation Study

Three ablation experiments systematically evaluated the component contributions:

(1) The acoustic baseline SLKNet without knowledge distillation.
(2) The vibration–acoustic knowledge transfer method KD-AV employing basic distillation.
(3) The proposed joint knowledge distillation method JKD-AV.

**Figure 12:**
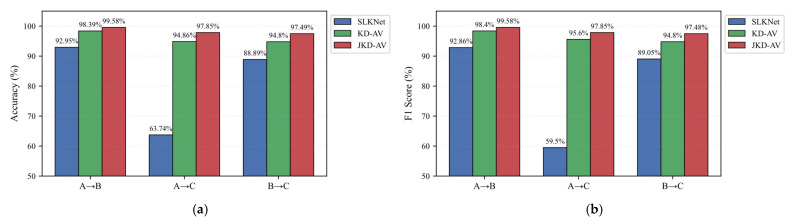
Ablation study results. (**a**) Accuracy; (**b**) F1-score.

All methods maintained identical network parameters with only the distillation strategies varied to ensure comparability. The results in Table 3 and Figure 12 demonstrate the consistent superiority of distillation techniques in cross-domain diagnosis.

Compared to SLKNet, KD-AV improved the accuracy by 5–30% across tasks, achieving 31.12% accuracy and 36.10% F1-score gains in the challenging A → C task, confirming vibration signals’ critical role in knowledge transfer. Crucially, JKD-AV attained 97.85% accuracy in A → C, surpassing basic distillation by 2.99%, while maintaining a minimum performance above 97.49% across all tasks. This validates the efficacy of jointly distilling intermediate and output layer features. The JKD-AV delivered optimal performance consistently, excelling in stable high-precision execution on challenging Task A-C while eliminating performance fluctuations in Task B → C. These results conclusively demonstrate the vibration signals’ enhancement effect on the acoustic diagnosis.

#### 4.1.4. Comparative Experiments

Given limited prior work on cross-modal feature transfer between acoustics and vibration, three critical comparisons were designed:

(1) Support vector machines’ processing of acoustic signals established SLKNet’s advantage over classical machine learning.
(2) The maximum mean discrepancy alignment for acoustic signals served as a feature transfer baseline to investigate noise interference.
(3) The MMD-based vibration-to-acoustic transfer provided a benchmark for conventional distribution alignment.

**Figure 13:**
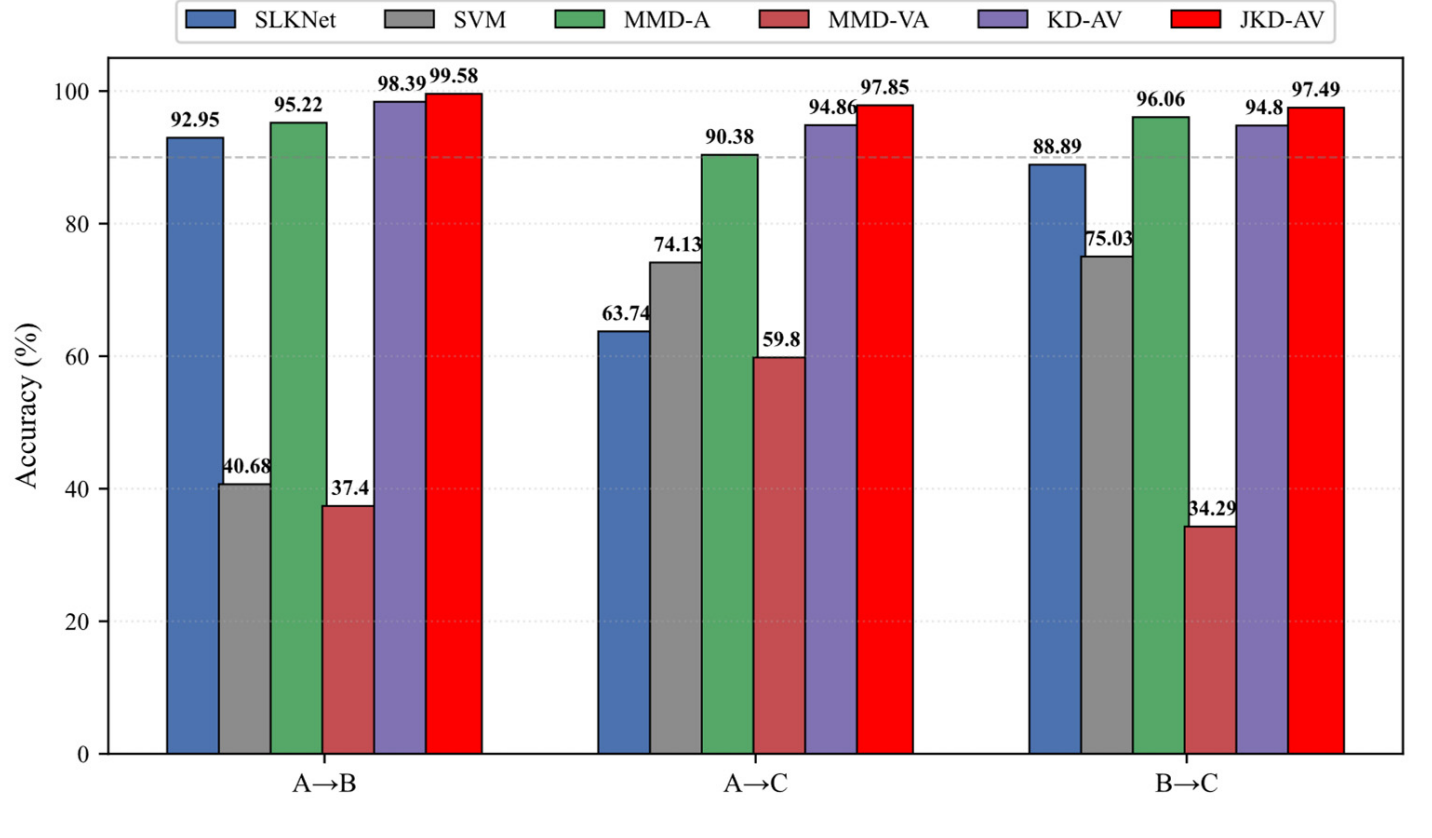
Comparative experimental results.

The results in Table 4 and Figure 13 show SVM achieved only 40.68% accuracy in A → B—52.27% lower than SLKNet’s 92.95%, confirming SLKNet’s superior generalization despite limited parameters. MMD-A outperformed other comparative models due to smaller homogeneous signal distribution shifts but declined significantly in the distributionally divergent A → C, lagging the KD-AV by 4.48% and JKD-AV by 7.47%. This verifies acoustic noise degrades cross-domain generalization. Conversely, the MMD-VA performed worst across all tasks, with A → B accuracy 37.40%, A → C 59.80%, and B → C 34.29%—substantially below SLKNet’s corresponding 92.95%, 63.74%, and 88.89%. This indicates direct MMD-based vibration–acoustic alignment induces negative transfer. Ultimately, the KD-AV and JKD-AV demonstrated superior performance. Notably, KD-AV’s 94.80% accuracy in B → C was lower than MMD-A’s 96.06%, revealing the limitations of output-layer distillation. The proposed JKD-AV achieved optimal results comprehensively: 99.58% for A → B, 97.85% for A → C, and 97.49% for B → C, validating the efficacy of cross-modal joint knowledge distillation.

### 4.2. Case Study II: BJTU-RAO Bogie Dataset

#### 4.2.1. Dataset Description

**Figure 14:**
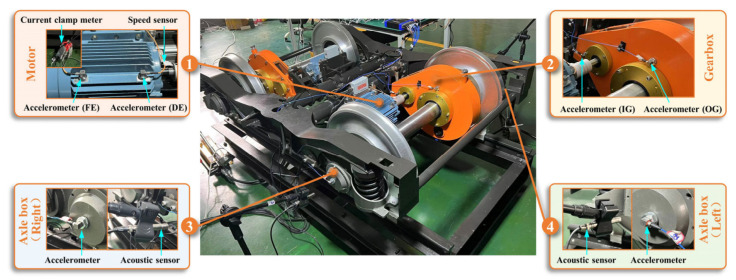
Schematic of the bogie transmission test rig (adapted from [40]).

This study employs the BJTU-RAO bogie dataset [40] released by Beijing Jiaotong University’s National Key Laboratory of Advanced Rail Transit Autonomous Operation for validation. As the first open-source dataset globally targeting the fault diagnosis of rail transit train bogie transmission systems, it was generated using a 1:2 scaled fault simulation test rig for metro train bogie transmissions. As illustrated in Figure 14, the rig’s core drivetrain comprises a three-phase induction motor, reduction gearbox, and axle boxes. It simulates multiple operating conditions including variable speeds and lateral loads, encompassing 51 health states spanning component-level single/composite faults and system-level correlated compound faults. The sensor deployment replicates actual vehicle measurement points.

To validate the proposed fault diagnosis method, a variable-load transfer experiment was designed. A critical subset of faults in the left motor-end axle box bearings (model: HRB 352213) from the BJTU-RAO dataset was selected, comprising four distinct health conditions: inner race faults, outer race faults, roller element faults, and normal states. Under a constant motor speed of 60 Hz, this subset focuses on two typical conditions: 0 kN lateral load simulating straight-line operation and −10 kN simulating curving conditions. Subset data include triaxial accelerometer vibration signals using x-axis channel data and single-channel acoustic signals from microphones, all sampled at 64 kHz. The 0 kN load condition serves as the training set; the −10 kN data are split 1:9 into validation and test sets. The validation set tunes the model hyperparameters, while the test set assesses the generalization under unseen conditions via transfer learning.

#### 4.2.2. Experimental Results

**Figure 15:**
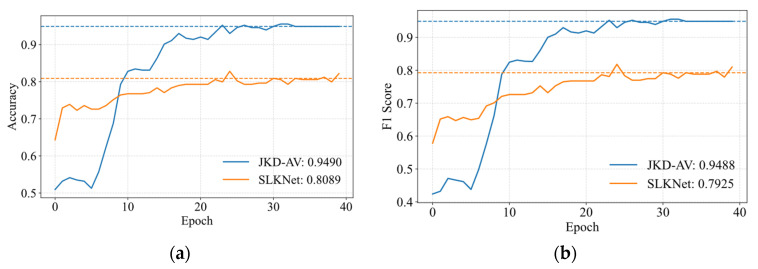
Optimization convergence characteristics for task 0 kN → −10 kN transfer. (**a**) Validation accuracy; (**b**) F1-score.

To systematically validate the method’s generalizability, the proposed approach was tested on the publicly available dataset for the 0 kN to −10 kN transfer task under more complex load conditions. Figure 15 demonstrates the steep ascent of JKD-AV’s validation accuracy curve, achieving a peak of 94.90%—14.01 percentage points higher than the SLKNet baseline. The dashed lines in the figures represent the average results of the last five epochs on the validation set during training, and their specific values are shown in the legend. This fully demonstrates the effectiveness of knowledge distillation in improving the generalization ability of complex equipment model diagnosis.

**Figure 16:**
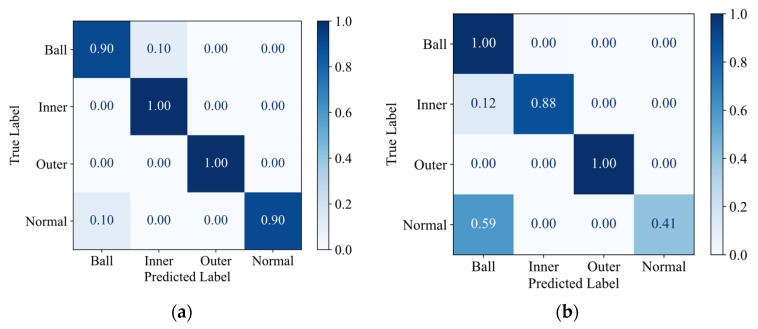
Confusion matrices for task 0 kN → −10 kN transfer. (**a**) JKD-AV; (**b**) SLKNet.

This advantage is further evidenced in the optimization of classification boundaries. Comparative analysis in Figure 16 reveals that the JKD-AV significantly reduces critical misclassifications, limiting the mutual misclassification rate between healthy states and rolling element faults to 10%. In stark contrast, SLKNet exhibits substantial misclassification errors: a notably high 59% mutual confusion rate between healthy states and rolling element faults, compounded by 12% of inner race faults being misclassified as rolling element faults.

**Figure 17:**
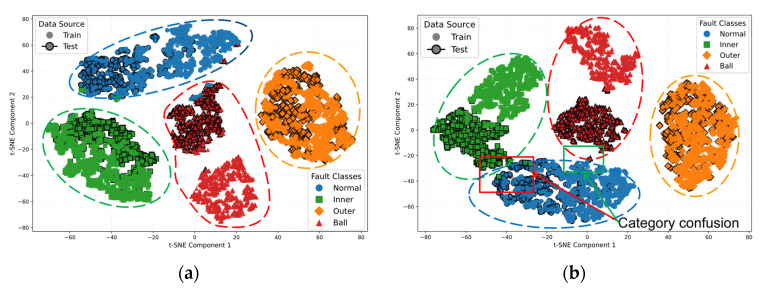
t-SNE visualizations for task 0 kN → −10 kN transfer. (**a**) JKD-AV; (**b**) SLKNet.

This stability improvement originates from the structural refinement of the feature space. Figure 17 visually characterizes the feature distributions via t-SNE. Figure 17a confirms the JKD-AV maintains training–test feature consistency despite complex loads; dispersion in ball faults does not compromise the class separability. Conversely, Figure 17b shows SLKNet suffering from expanded overlap between healthy and inner race faults, with additional confusion involving ball faults, revealing inherent deficiencies in noise immunity.

#### 4.2.3. Comparative Experiments

**Figure 18:**
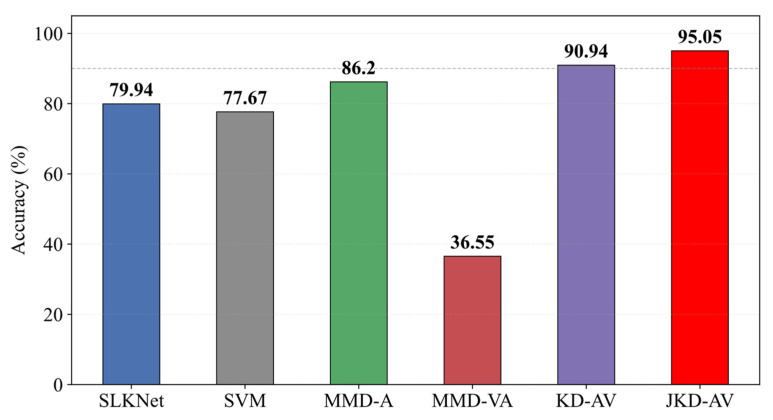
Comparative experimental results.

The comparative experimental analysis in Figure 18 shows the JKD-AV achieving 95.05% accuracy, significantly outperforming the homogeneous-signal alignment method MMD-A (86.20%) by an 8.85% margin. This verifies the indispensability of the vibration signal assistance. The MMD-VA method attained only 36.55% accuracy with evident negative transfer, fully exposing the inherent limitations of conventional distribution alignment in cross-signal scenarios. Under structurally more complex equipment and demanding load conditions, JKD-AV maintains robust 95.05% accuracy, conclusively demonstrating its strong generalization capability and engineering applicability.

### 4.3. Deployment of Models to Embedded Devices

**Figure 19:**
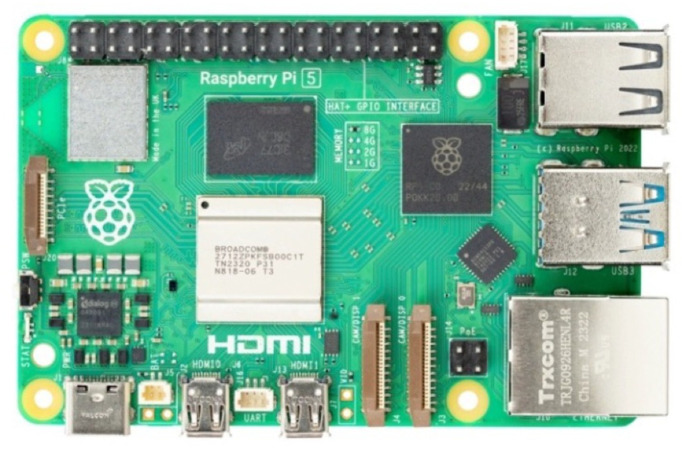
Raspberry Pi pictures.

To validate the feasibility of deploying the proposed SLKNet model on embedded devices, experimental verification was conducted on a Raspberry Pi 5B platform. The Raspberry Pi 5B is a single-board computer running a Linux-based operating system, equipped with 4 GB RAM and an 800 MHz VideoCore VII GPU. Its versatile expansion and I/O interfaces enable the integration of acoustic signal acquisition, data preprocessing, and deep learning model inference within a unified system. A physical image of the Raspberry Pi is shown in Figure 19.

As shown in Table 5, the SLKNet model exhibits a compact architecture with a size of 0.03 MB and an exceptionally low parameter count of 325. When deployed on a Raspberry Pi platform for inference testing on 1860 samples from Case 1 Dataset C, the total processing time was merely 0.25 s, yielding an average per-sample inference latency as low as 134 μs. This result demonstrates that the distilled student model achieves exemplary inference efficiency, fully satisfying the real-time requirements for edge device deployment in industrial fault diagnosis scenarios.

## 5. Conclusions and Future Work

Addressing the highly challenging and relatively understudied problem of acoustic–vibration fusion and cross-modal knowledge transfer, this paper introduces a novel feature-level fusion and transfer strategy, proposing a joint knowledge distillation-based acoustic–vibration knowledge transfer diagnosis method (JKD-AV). Distinct from existing approaches based on fine-tuning or data generation, this method bridges the feature discrepancies caused by homologous yet heterogeneous characteristics through knowledge transfer technology that fuses shared knowledge between vibration and acoustic signals. It effectively enhances the diagnostic robustness and generalization capability of acoustic models under noise interference and variable load conditions. The experimental results demonstrate a diagnostic accuracy exceeding 95% across four designed transfer tasks. Compared to methods utilizing solely acoustic signals or conventional vibration–acoustic cross-signal transfer, the proposed method exhibits significant performance advantages while simultaneously providing a novel solution perspective for vibration–acoustic cross-modal knowledge transfer. This advancement further delivers an innovative approach for non-contact monitoring of industrial equipment. This achievement holds significant engineering value for real-time monitoring of critical equipment in smart manufacturing and simultaneously offers a reference basis for the application expansion of cross-modal transfer learning theory in industrial scenarios.

Despite the effectiveness of the proposed method in acoustic–vibration signal transfer diagnosis, key challenges warrant further exploration. First, the diagnostic capability of the teacher model is inherently constrained by the limited scale of vibration samples, where knowledge completeness depends heavily on the source domain data scale. Future research will establish a multi-source equipment vibration database. Training multi-teacher models will aggregate fault feature priors across diverse operational conditions to enhance generalization in complex scenarios. Second, while joint intermediate-output layer feature alignment via knowledge distillation proves effective, explicit modeling within the feature space of the physical discrepancy between signal generation mechanisms—specifically, the structural response captured in vibration signals versus airborne propagation governing acoustic signals—remains insufficient. To address this, we intend to introduce physics-informed neural networks, incorporating physical constraints underlying acoustic and vibration propagation, to develop more physically interpretable and efficient knowledge transfer strategies.

## Figures and Tables

**Table 1:** Composition of self-collected dataset by working condition and health state.

Dataset	Health States	Label	Working Conditions	Number of Single-Mode Samples
A	Ball fault	0	30 Hz_0 Nm	1860
	Inner race fault	1		
	Outer race fault	2		
	Normal	3		
B	Ball fault	0	30 Hz_3 Nm	1860
	Inner race fault	1		
	Outer race fault	2		
	Normal	3		
C	Ball fault	0	30 Hz_6 Nm	1860
	Inner race fault	1		
	Outer race fault	2		
	Normal	3		

**Table 2:** Configuration of transfer learning tasks.

Transfer Task	Training Set		Validation and Test Sets
	Source Domain (Vibration)	Target Domain (Acoustic)	
A → B	Dataset A	Dataset A	Dataset B
A → C	Dataset A	Dataset A	Dataset C
B → C	Dataset B	Dataset B	Dataset C

**Table 3:** Ablation study results.

Method	A → B		A → C		B → C	
	Accuracy	F1-Score	Accuracy	F1-Score	Accuracy	F1-Score
SLKNet	92.95%	92.86%	63.74%	59.50%	88.89%	89.05%
KD-AV	98.39%	98.40%	94.86%	95.60%	94.80%	94.80%
JKD-AV	99.58%	99.58%	97.85%	97.85%	97.49%	97.48%

**Table 4:** Comparative experimental results.

Method	A → B		A → C		B → C	
	Accuracy	F1-Score	Accuracy	F1-Score	Accuracy	F1-Score
SLKNet	92.95%	92.86%	63.74%	59.50%	88.89%	89.05%
SVM	40.68%	37.23%	74.13%	64.49%	75.03%	66.46%
MMD-A	95.22%	95.15%	90.38%	90.41%	96.06%	96.05%
MMD-VA	37.4%	33.42%	59.80%	54.10%	34.29%	25.26%
KD-AV	98.39%	98.40%	94.86%	95.60%	94.80%	94.80%
JKD-AV	99.58%	99.58%	97.85%	97.85%	97.49%	97.48%

**Table 5:** Model size and model testing time.

Model Size	Total Params	Test Time (Single Sample)/μs
0.03 M	325	134

## Data Availability

The data presented in this study are available upon request from the corresponding author. The data are not publicly available due to privacy concerns.
